# Improved Prediction of Falls in Community-Dwelling Older Adults Through Phase-Dependent Entropy of Daily-Life Walking

**DOI:** 10.3389/fnagi.2018.00044

**Published:** 2018-03-05

**Authors:** Espen A. F. Ihlen, Kimberley S. van Schooten, Sjoerd M. Bruijn, Jaap H. van Dieën, Beatrix Vereijken, Jorunn L. Helbostad, Mirjam Pijnappels

**Affiliations:** ^1^Department of Neuromedicine and Movement Science, Norwegian University of Science and Technology (NTNU), Trondheim, Norway; ^2^Department of Biomedical Kinesiology and Physiology, Simon Fraser University, Burnaby, BC, Canada; ^3^Burnaby and Centre for Hip Health and Mobility, University of British Columbia, Vancouver, BC, Canada; ^4^Department of Human Movement Sciences, Vrije Universiteit, Amsterdam, Netherlands

**Keywords:** accelerometry, complexity, gait assessment, physical activity, aged, fall prediction, fall risk, accidental falls

## Abstract

Age and age-related diseases have been suggested to decrease entropy of human gait kinematics, which is thought to make older adults more susceptible to falls. In this study we introduce a new entropy measure, called phase-dependent generalized multiscale entropy (PGME), and test whether this measure improves fall-risk prediction in community-dwelling older adults. PGME can assess phase-dependent changes in the stability of gait dynamics that result from kinematic changes in events such as heel strike and toe-off. PGME was assessed for trunk acceleration of 30 s walking epochs in a re-analysis of 1 week of daily-life activity data from the FARAO study, originally described by van Schooten et al. (2016). The re-analyzed data set contained inertial sensor data from 52 single- and 46 multiple-time prospective fallers in a 6 months follow-up period, and an equal number of non-falling controls matched by age, weight, height, gender, and the use of walking aids. The predictive ability of PGME for falls was assessed using a partial least squares regression. PGME had a superior predictive ability of falls among single-time prospective fallers when compared to the other gait features. The single-time fallers had a higher PGME (*p* < 0.0001) of their trunk acceleration at 60% of their step cycle when compared with non-fallers. No significant differences were found between PGME of multiple-time fallers and non-fallers, but PGME was found to improve the prediction model of multiple-time fallers when combined with other gait features. These findings suggest that taking into account phase-dependent changes in the stability of the gait dynamics has additional value for predicting falls in older people, especially for single-time prospective fallers.

## Introduction

Falls among older persons are an important cause of loss of independence, reduced quality of life, and admission to hospitals or nursing homes. At a European level, the annual costs of falls among older persons are estimated to be 30 billion euro (Stevens et al., 2006). Early prediction of falls amongst community-dwelling older persons would provide increased opportunities for fall prevention.

More than 400 risk factors for falls have been reported (e.g., Deandrea et al., 2010). Most risk factors have been assessed in laboratory settings or in clinical test situations, and fall risk assessment systems have been developed to serve as screening tools for fall risk (Oliver et al., 1997; Raîche et al., 2000; Tromp et al., 2001; Nandy et al., 2004). Several clinical tests of balance and mobility performance, such as the Timed Up and Go, sit-to-stand and alternate step tests, have been suggested as part of fall risk screening tools (Bohannon, 2006; Tiedemann et al., 2010). However, most of these clinical tests and screening tools suffer from ceiling effects, reflect the performance of the older person at a specific moment in time, or are based on self-reports (Cofre et al., 2014). Falls in older persons are experienced during activities of daily life, most frequently during walking or in transitions to walking (Becker et al., 2012; Robinovich et al., 2013). Accordingly, metrics of daily-life walking have been shown to improve early fall prediction in community-dwelling older persons beyond the ability of clinical screening tools for fall risk (Weiss et al., 2013; Rispens et al., 2015; van Schooten et al., 2016). These metrics can be assessed by analysis of 3D acceleration data from body-worn sensors that could be self-managed by the older adults through smart technology (Shany et al., 2012).

Aging is considered to be related to reduction of complexity and irregularity in multiple physiological systems (Lipsitz and Goldberger, 1992), suggesting reduced ability of physiological processes to adapt to various stressors. More specifically, loss of irregularity in movement variability in daily-life walking could be a predictor of falls, as it reflects an older person's inability to adapt to perturbations in their daily-life environment. Previous studies have shown that stability and complexity metrics of trunk acceleration signals of daily-life walking, such as Lyapunov exponents and entropy measures, improve fall prediction compared to conventional measures such as the amount and intensity of the walking activity (Rispens et al., 2015; van Schooten et al., 2016).

In addition to improving fall risk assessment based on complexity and stability metrics, spectral analysis suggests that the information important for fall risk assessment is contained in frequency ranges of >2.5 Hz of the trunk acceleration signal, indicating changes in the intra-step modulation of the trunk acceleration (Weiss et al., 2013). However, the limitation of spectral analysis is the lack of a direct relationship to the stability and complexity metrics assessed in nonlinear analyses of the trunk dynamics. Moreover, few of the nonlinear and spectral analyses yield phase-dependent metrics that are able to assess changes in the structure of signal variability within the gait cycles (Ihlen et al., 2015). Combining spectral analysis and analysis of stability and irregularity is important to develop new measures for more precise fall prediction models. Thus, in this study we aimed to introduce a new entropy measure of daily-life walking, called phase-dependent generalized multiscale entropy (PGME), that combines nonlinear analysis, spectral decomposition, and phase-dependent analysis of the trunk acceleration data. Subsequently, we tested whether including these features improved the prediction of falls in community-dwelling older adults.

## Methods

### Participants and data collection

Inertial sensor data, previously reported by van Schooten et al. (2016), were re-analyzed in the present study (see acknowledgments). The data consisted of 1 week of accelerometer data at baseline from 303 community-dwelling older persons, and prospective fall assessment by monthly telephone interviews in a 12-month follow-up period. Fifty-eight participants experienced a single fall and 46 two or more falls (multiple falls) in the 6-month follow-up period that was considered in the present study. Single- and multiple-time fallers were matched by gender, age, weight, height, and use of walking aids to the remaining 199 non-fallers. Summary of the selected participants' characteristics is provided in Table 1. The 3D-acceleration was sampled at 100 Hz by a small inertial sensor worn with a belt over the lower back (DynaPort Hybrid, McRoberts, The Hague, Netherlands; 87 × 45 × 14 mm, 74 g); the sensor had a range and resolution of ±6 g and ±1 mg, respectively. This study was carried out in accordance with the recommendations of the medical ethical committee of the VU Medical Hospital (protocol 2010/290). All subjects provided written informed consent in accordance with the Declaration of Helsinki. The protocol was approved by the medical ethical committee of the VU Medical Hospital. The reader is referred to van Schooten et al. (2016) for further details about participants and protocols.

**Table 1:** Demographic variables and clinical tests of the single- and multiple time fallers and their matched non-fallers.

	**Fallers**	**Matched non-fallers**	*p*-value^*^
**SINGLE-TIME FALLERS**			
Gender (% female)	51	51	1
Age (years, mean ± SD)	76.1 ± 6.8	75.9 ± 6.7	0.91
Height (cm, mean ± SD)	170.8 ± 9.2	170.6 ± 8.3	0.74
Weight (kg, mean ± SD)	73.2 ± 12.9	72.7 ± 12.3	0.33
Assisted living (%)	3.8	5.6	0.58
Residential care (%)	1.9	3.8	0.48
Walking aid (%)	18.9	18.9	1
MMSE (median/range)	28/9	28/9	0.85
≥1 falls in past 6 months (%)	47.2	34.0	0.05
**MULTIPLE-TIME FALLERS**			
Gender (%)	48.8	48.8	1
Age (yrs, mean±SD)	75.5 ± 6.7	75.2 ± 6.4	0.99
Height (cm, mean±SD)	170.9 ± 8.2	171.6 ± 7.8	0.16
Weight (kg, mean ± SD)	75.6 ± 10.8	74.6 ± 10.8	0.04
Assisted living (%)	9.8	4.9	0.12
Residential care (%)	0	0	1
Walking aid (%)	17.1	17.1	1
MMSE (mean ± SD)	28/10	28/9	0.82
≥1 falls in past 6 months (%)	53.7	34.2	0.005

* *Note that the p-values are given for a one-sample t-test or Wilcoxon signed rank test depending on the distribution of the data*.

### Preparation of data

The following procedure was used to identify daily-life walking: First, the 3D accelerations for walking bouts with ≥3 s duration were identified by a commercially available activity detection algorithm (McRoberts BV, the Hague, Netherlands). Secondly, 3D accelerations for walking bouts with duration of ≥30 s were included in further analysis. Thirdly, all included walking bouts were converted into equal sized 30 s epochs (i.e., 3,000 samples) to provide a consistent sample size for computation of entropy measures. Fourthly, all included epochs were visually checked and non-walking activity was excluded. Inclusion criteria for walking epochs were: (1) distinct impact peaks in vertical (V) and/or anterioposterior (AP) direction of the acceleration signal, (2) distinct cyclical acceleration pattern in V and/or AP direction, and (3) criteria 1 and 2 were satisfied for at least 80% of the epoch, where max 20% was considered as gait initiation, turning or transitional micro-breaks. These inclusion criteria were developed from visual inspection of fast, normal and slow walking from a validation study for activity detection in older persons (Bourke et al., 2017). Seventy-one percent of the total of 59762 epochs of ≥30 s were considered walking epochs and were included in the further analysis.

The step cycle was identified through the following procedure: First, the 3D-velocity was estimated by numerical integration of the detrended acceleration signals. Secondly, the 3D-velocity signals were detrended using a multivariate empirical mode decomposition (MEMD) procedure that preserved intra-step variation but removed inter-stride nonlinear trends (see Appendix A for technical details for MEMD). Thirdly, the local minima of the vertical velocity were identified and the step-cycle considered as the interval between consecutive minima.

### Phase-dependent multiscale *q*-order entropy analysis

Entropy defines the average level of irregularity in a time series. The irregularity of the dynamics of a time series can be defined by the Kolmogorov-Sinai entropy, but estimation of this metric requires long time series with a low level of noise, which cannot be obtained in physiological time series (Kolmogorov, 1958; Sinai, 1959; Kantz and Schreiber, 2004). More recently, approximate entropy (Pincus, 1991), sample entropy (Richman and Moorman, 2000) and permutation entropy (Bandt and Pompe, 2002) have been proposed as measures of irregularity of short, noisy time series from physiological processes. Multiscale extensions of both sample entropy and permutation entropy have been developed, which are able to assess the irregularity of time series at multiple scales or frequencies (Costa et al., 2002; Li et al., 2010; Wu et al., 2014). The multiscale entropy makes it possible to investigate the high frequency intra-step modulation of trunk acceleration signals of walking. However, all entropy metrics above have the following fundamental shortcomings when applied to physiological processes: (1) they do not decompose the dynamics into multiple scales or do so based on multiple coarse grained versions of the dynamics, making the results difficult to interpret because no unique version of the dynamics is analyzed. (2) they assume that the entropy metrics are invariant to phase transitions common in physiological processes such as daily life walking, although there is no empirical validation for this assumption. (3) they are based on the Boltzman-Gibbs-Shannon concept of entropy, which assumes that no phase transitions and emerging behavior are present (Shannon, 1948), although phase-transitions and emergent behaviors through complex interactions of sub-systems are a rule rather than an exception in physiological processes such as daily-life walking (Ihlen et al., 2017).

**Figure 1:**
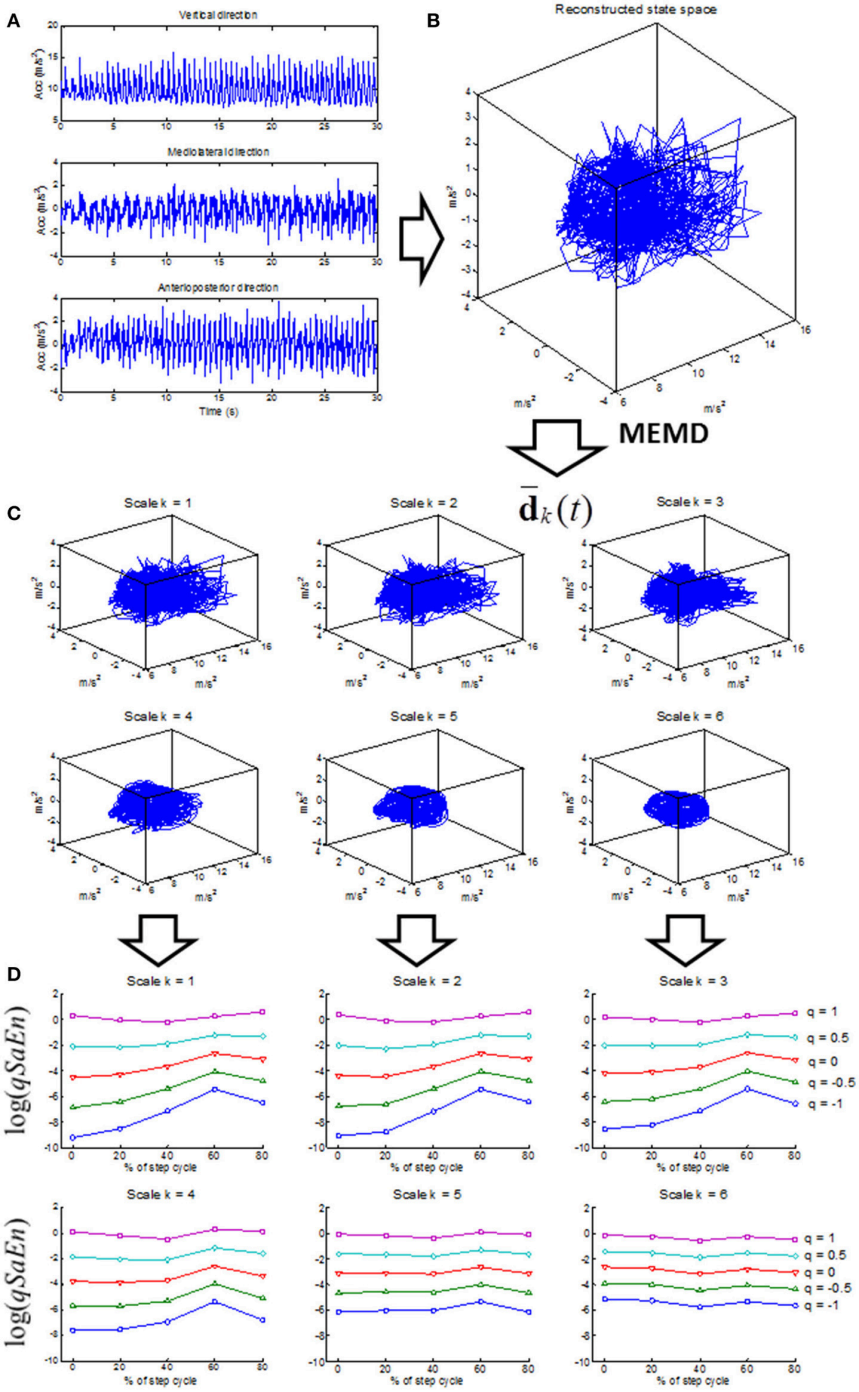
Flow chart of the computation of phase-dependent generalized multiscale entropy (PGME). The 3D trunk acceleration signal **(A)** is used to reconstruct the gait dynamics **(B)** according to Equation (1, 2). Note that only three-dimensional state spaces are displayed in this flow chart even though the state spaces in Equation (1, 2) are six and nine dimensional. The reconstructed gait dynamics is decomposed by multivariate empirical mode decomposition (MEMD) where the low-pass filtered versions ${\bar{d}}_{k}(t)$ of the gait dynamics (Equation 3) are displayed in the **(C)** for scale *k* = 1 to 6 (further technical details in Appendix A in Supplementary Material). The generalized sample entropy (qSaEn) is computed for each scale by Eq. 4 and 5 (further technical details in Appendix B in Supplementary Material) for phase 0, 20, 40, 60, and 80% of the step cycle. The **(D)** display qSaEn in log-coordinates for scale k = 1 to 6 and each panel displays qSaEn as a function of phase of the step cycle for *q* = [−1, −0.5, 0, 0.5, 1] (different colored traces).

The present study introduces a phase-dependent generalized multiscale entropy (PGME) that solves the shortcomings described above. PGME is defined by the following four-step procedure for the analysis of the 3D acceleration data of daily-life walking (see flow chart in Figure 1):

#### Reconstruction of trunk dynamics

The trunk dynamics during daily-life walking are reconstructed by the 3D acceleration data for the included 30 s walking epochs, as follows:

(1) $$X_{j}=\left[a_{j}^{AP},a_{j}^{ML},a_{j}^{V},a_{j+l}^{AP},a_{j+l}^{ML},a_{j+l}^{V}\right]$$

(2) $$Y_{j}=\left[a_{j}^{AP},a_{j}^{ML},a_{j}^{V},a_{j+l}^{AP},a_{j+l}^{ML},a_{j+l}^{V},a_{j+2l}^{AP},a_{j+2l}^{ML},a_{j+2l}^{V}\right]$$

where *a* is the acceleration signal in anteriorposterior (AP), mediolateral (ML), and vertical (V) directions, and where the subscript *j* is the time sample index. The lag size *l* in Equations (1, 2) was estimated for each 30-s epoch by the inflection point in the average mutual information (AMI) function, where AMI decreased with >0.01 per sample. These choices of state spaces have been shown to optimize the classification of fallers and non-fallers in a previous study (Ihlen et al., 2016b).

#### Multivariate empirical mode decomposition (MEMD) of the trunk dynamics

The trunk dynamics reconstructed in step 1 are decomposed by MEMD into multiple intrinsic mode functions (IMFs) by a five-step iterative shifting procedure (see Appendix A for further technical details). IMFs are defined in an iterative way from high to low frequency modes, where the frequency range is dependent on intrinsic properties of the dynamics. Thus, the IMFs are more related to the physiological process than conventional Fourier functions with their predefined frequency ranges. In contrast to a univariate EMD, MEMD is able to detect common intrinsic modes across all dimensions of the reconstructed dynamics (i.e., AP, ML, and V directions of the acceleration signal). The coarse-grained versions ${\bar{d}}_{k}(t)$ of the trunk dynamics were estimated as the following sum of IMFs:

(3) $${\bar{d}}_{k}(t)=\sum_{k}^{M}d_{k}(t)$$

where **d**_*k*_(*t*) is the IMF for scale *k* ≤ *M, M* is the total number of IMFs and *k* = 1, 2, …, *M* is the high to low frequency ordering of the **d**_*k*_(*t*). The coarse-grained versions ${\bar{d}}_{k}(t)$ for *k* = 1 return the original dynamics and *k* > 1 their low-pass filtered versions. In the present study, ${\bar{d}}_{k}(t)$ for *k* = 1, 2, …, 6 was considered because these versions contained the phase-dependent details of the dynamics at frequencies higher than the main periodicity of the step cycle of about 2 Hz. The ${\bar{d}}_{k}(t)$ of a 3D reconstructed state space are illustrated in Figure 1C.

#### Generalized sample entropy (qSaEn)

A generalized sample entropy (qSaEn) was computed for each of ${\bar{d}}_{k}(t)$ defined by Equation (3). qSaEn is the *q*-logarithm of the probability that for two data points in the reconstructed state space of Equation (1) with a distance below *r*. Their equivalent points in the reconstructed state space of Equation (2) also have a distance below *r*. qSaEn was defined by the following equation (Silva and Murta, 2012):

(4) $$qSaEn_{r}=\log_{q}(n_{x})-\log_{q}(n_{y})$$

where the *n*_*x*_ and *n*_*y*_ are the total normalized number of points in state space with distance below *r* of the reconstructed gait dynamic of Equation (1, 2), respectively, across all *i* and *j* points in the state space where *i* ≠ *j* (see Equations B1–B5 in Appendix B for technical details). The *q*-order logarithm was defined by the following two functions for *n*_x_ and *n*_y_, respectively:

(5) $$\log_{q}(n_{x})=\frac{n_{x}^{1-q}-1}{1-q}\;\log_{q}(n_{y})=\frac{n_{y}^{1-q}-1}{1-q}$$

**Figure 2:**
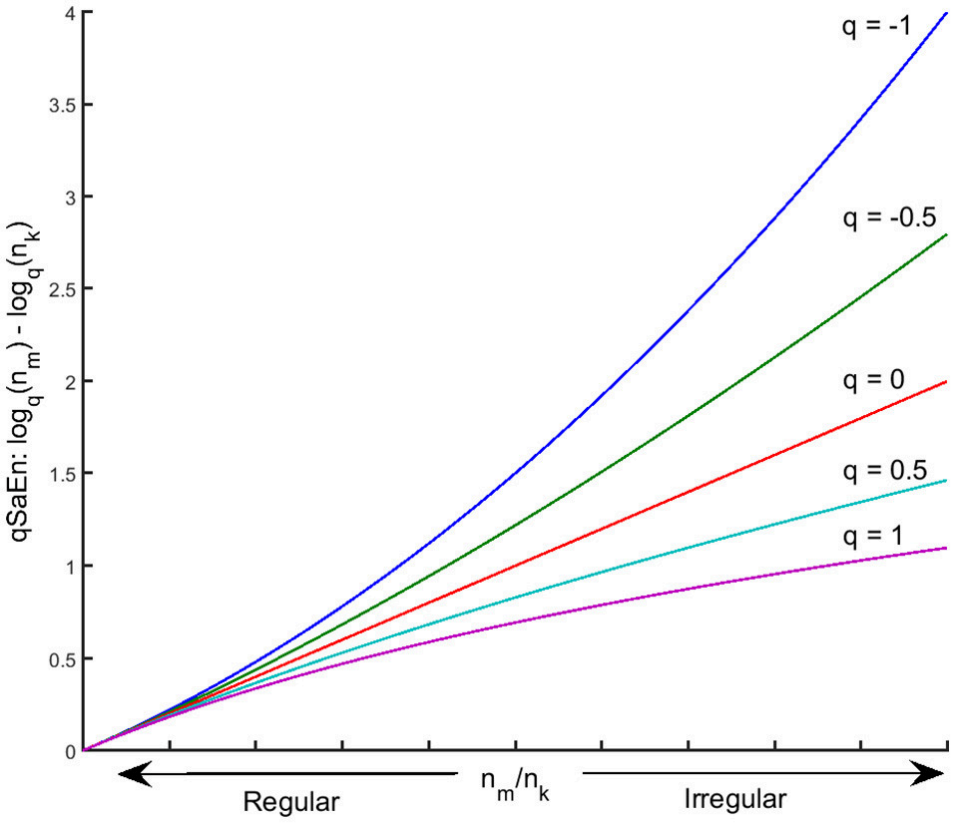
Plot of qSaEn in Equation (4) as a q-order log-function of the count ratio, *n*_*m*_/*n*_*k*_ for *q* = −1, −0.5, 0, 0.5, and 1. The *q*-order log-function amplifies the larger *n*_*m*_/*n*_*k*_ (i.e., larger irregularity) for *q* < 0 (blue and green line) whereas it penalize the large *n*_*m*_/*n*_*k*_ for *q* > 0 (light blue and violet line).

Tsallis (1988) introduced the *q*-order logarithm in Equation (5) above as an extension of the logarithm in the conventional Boltzman-Gibbs-Shannon entropy to quantify entropy in complex systems with inhomogeneous dynamics due to phase transitions. Values of *q* below 0 penalize high probabilities that two data points have the same distance < *r* for both reconstructed state space of Equations (1, 2), whereas values of *q* above 0 penalize low probabilities for the same situation. Consequently, *q* < 0 penalizes low information loss (i.e., regular signals) whereas *q* > 0 penalizes high information loss (i.e., irregular signal) (see Figure 2). The walking dynamics have been shown to be phase-dependent where intrinsic properties of the walking dynamics change within the gait cycle. The *q*-order logarithm in qSaEn has the ability to penalize the level of irregularity in different phases of the gait cycle, like toe-off and heel strikes, thereby detecting phase-dependent changes in irregularity not possible by the conventional sample entropy. In the present study, we used parameter setting *r* = 0.3 SD, where SD was considered as the mean standard deviation across AP, ML, and V directions, following a procedure suggested by Lake et al. (2002) based on the minimum relative error in the PGME estimate. Furthermore, a *q*-range of −1 to 1 was chosen for qSaEn based on previous studies on generalized sample entropies (Silva and Murta, 2012). Further technical details on the computation of qSaEn are found in Appendix B.

#### Phase-dependent qSaEn

The phase-dependent qSaEn was defined by Eq. 4 across samples *j* within 10% of the step time after 0, 20, 40, 60, and 80% of each step cycle. The step cycle was identified by maxima of the vertical velocity and was comparable to results from step identification based on the autocorrelation of the acceleration signal (Moe-Nilssen and Helbostad, 2004). The Matlab code for calculation of the PGME, including steps 2–4 above, is provided in Appendix C.

### The predictive value of PGME for falls

The computation of PGME was performed on all included walking epochs after which the median PGME was computed across all epochs of each individual. The median PGME consisted of an entropy metric for each of 6 temporal scales, 5 phases of the step cycle and 21 *q* values. The ability of median PGME to predict single and multiple falls in a 6-month follow-up period was assessed by the following procedure:

#### Training and test data generation

Eighty percent of the data set was used as a training set, whereas 20% of the data set was used as a test set. A stratified holdout was used so that an equal proportion of fallers and non-fallers was included in the test and training sets. In addition, the pairs of matched fallers and non-fallers were kept in the same training or test set. In total 500 permutation test and training sets were generated to compute the confidence intervals for evaluation of the fall prediction model.

#### Feature subset generation

Three feature subsets were generated and compared: (1) PGME features, (2) gait features and demographic variables used in van Schooten et al. (2016), and (3) subsets 1 and 2 combined. Table 2 summarizes the gait features and demographic variables included in subset 2. All features in each of the subsets were normalized to Z-scores to prevent range-dependent influences of each feature.

**Table 2:** Gait features contained in feature set 2 used in the single- and multiple-time fall prediction model.

**Gait feature name**	**Direction**
Stride time	–
Walking speed	–
Walking distance	–
Mean step length	–
Stride time variability	–
Stride length variability	–
Stride speed variability	–
Stride frequency	–
Stride frequency variability	–
Acceleration standard deviation	AP, ML, V, Vector magnitude
Stride regularity (Moe-Nilssen and Helbostad, 2004)	AP, ML, V, Vector magnitude
Harmonic ratio (Yack and Berger, 1993; Doi et al., 2013)	AP, ML, V
Index of harmonicity (Lamoth et al., 2002)	AP, ML, V
Spectral range (Weiss et al., 2013)	AP, ML, V
Spectral dominant frequency (Weiss et al., 2013)	AP, ML, V
Spectral slope (Weiss et al., 2013)	AP, ML, V
Spectral width (Weiss et al., 2013)	AP, ML, V
Spectral amplitude (Weiss et al., 2013)	AP, ML, V
Low frequency percentage (Rispens et al., 2015)	AP, ML, V, Vector magnitude
Lyapunov exponent R (Rosenstein et al., 1993)	AP, ML, V, Vector magnitude
Lyapunov exponent W (Wolf et al., 1985)	AP, ML, V, Vector magnitude
Lyap. exponent per stride R (Rosenstein et al., 1993)	AP, ML, V, Vector magnitude
Lyap. exponent per stride W (Wolf et al., 1985)	AP, ML, V, Vector magnitude

#### Evaluation with partial least squares (PLS) regression

PLS regression was used to assess the ability of the three feature sets to predict single and multiple falls in the 6-month follow-up period. The PLS prediction model is able to identify a low-dimensional latent structure that predicts the fall status from a large number of gait features. In contrast to other regression approaches, PLS prediction models are designed to perform a prediction model based on a large set of noisy and collinear features without overfitting. Thus, the PLS prediction model is suitable for the large number of gait features investigated in the present study. The present study used a linear discriminant analysis (LDA) with an integrated non-linear iterative partial least squares (NIPALS) algorithm (Marigheto et al., 1998; Wold et al., 2001; Rosipal and Krämer, 2006). TP-loadings ranked the features within each of the three feature subsets based on their influence in the prediction model of single or multiple falls within the 6-month follow-up period (Kvalheim and Karstang, 1989).

A backward feature selection method was used for the PLS prediction model by the following four steps procedure: First, the error_*j*_ (i.e., 1–accuracy) was defined for the feature subset leaving out one of its features. The maximum number of latent vectors to search for in the response matrix was set at 10 vectors. Secondly, features for which exclusion caused the lowest error were removed from the feature subset creating a new reduced feature subset. Thirdly, the first and second steps were repeated until all features had been removed from the reduced subset. Fourthly, the reduced feature set that created the smallest error was selected in the final model. Finally, a 10-fold cross-validation was then used to evaluate the model performance.

#### Prediction model performance

The performance of the PLS fall prediction model was assessed for the 500 permutation test sets. The predictive ability of single- and multiple-time fallers in a 6-month follow-up period was assessed as the mean accuracy, sensitivity, specificity, positive predictive value, and negative predictive value. The 95% confidence intervals were computed by a bootstrap procedure (Efron and Tibshirani, 1986). To control for the different number of latent vectors in the PLS regression, the optimal fall prediction model across the three feature sets was obtained by the Akaike's information criterion for finite sample size (AICc). A significant difference ΔAICc = AICc_1_ − AICc_2_ was identified as a relative likelihood less than 0.05 (i.e., RL = exp([AICc_1_ − AICc_2_]/2) < 0.05) (Burnham et al., 2011). The prediction model's performance was also compared to the performance of 6 month fall history to gain insight into the added value of accelerometry over simple and commonly-used fall risk indicators. A Wilcoxon rank sum test with a Benjamini-Hochberg correction for multiple comparisons was performed to compare PGME metrics for each *q*, scale and step-phase (Benjamini and Hochberg, 1995). All statistical analyses in the present study were performed in Matlab version 2014a.

## Results

### Difference in PGME between fallers and non-fallers

**Figure 3:**
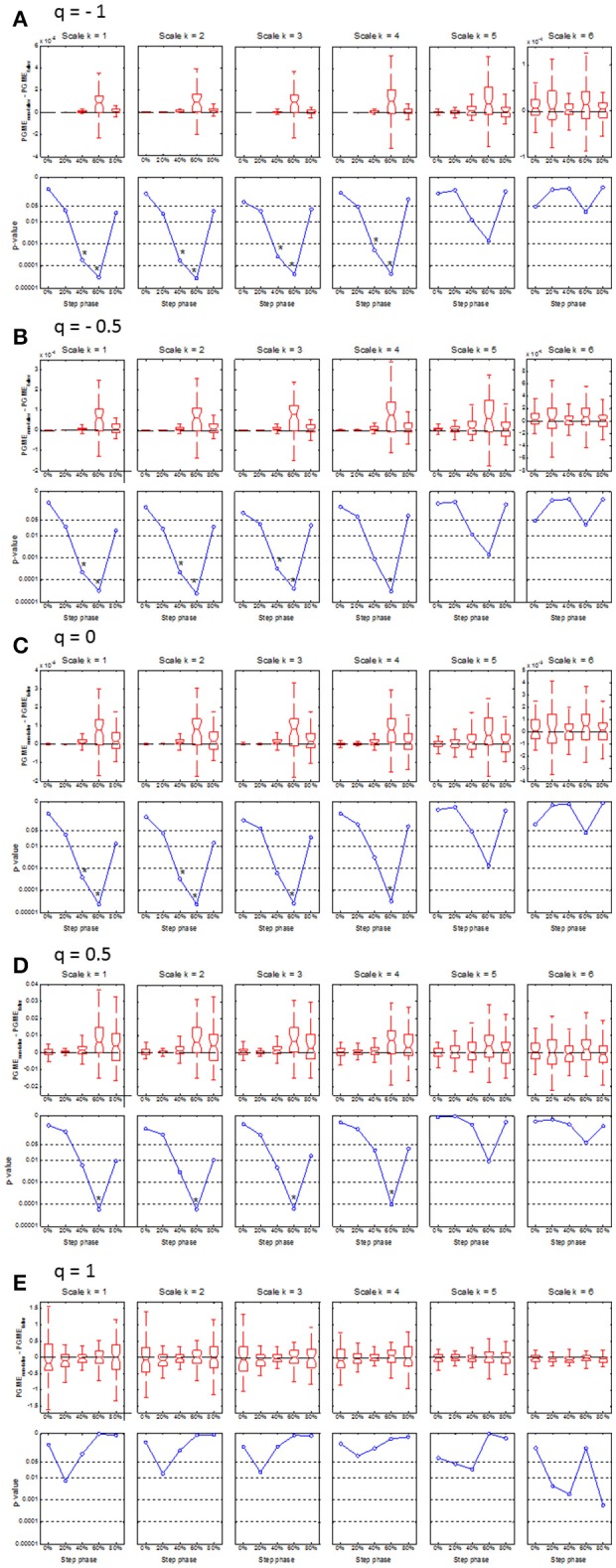
Boxplots (upper row) and corresponding *p*-values (lower row) of the median difference between PGME of single-time fallers and their matched non-falling controls. The x-axis of each plot indicates how the median difference or corresponding *p*-values changes with the step phase. The upper and lower panels on each row **(A**–**E)** indicates how the median difference and the corresponding *p*-values change with the scale *k* = 1 to 6. Rows **(A**–**E)** of the panels indicate how the median difference in PGME and corresponding *p*-values change according to *q*-orders, *q* = −1, −0.5, 0, 0.5 and 1, of the PGME. Note that the center of the boxes represents the group median and the upper and lower borders of the box represent the 75th and 25th percentile, respectively. The whiskers represent the most deviating values within 1.5 times the interquartile range from the median value and the notches represent the confidence interval of the median value.

**Figure 4:**
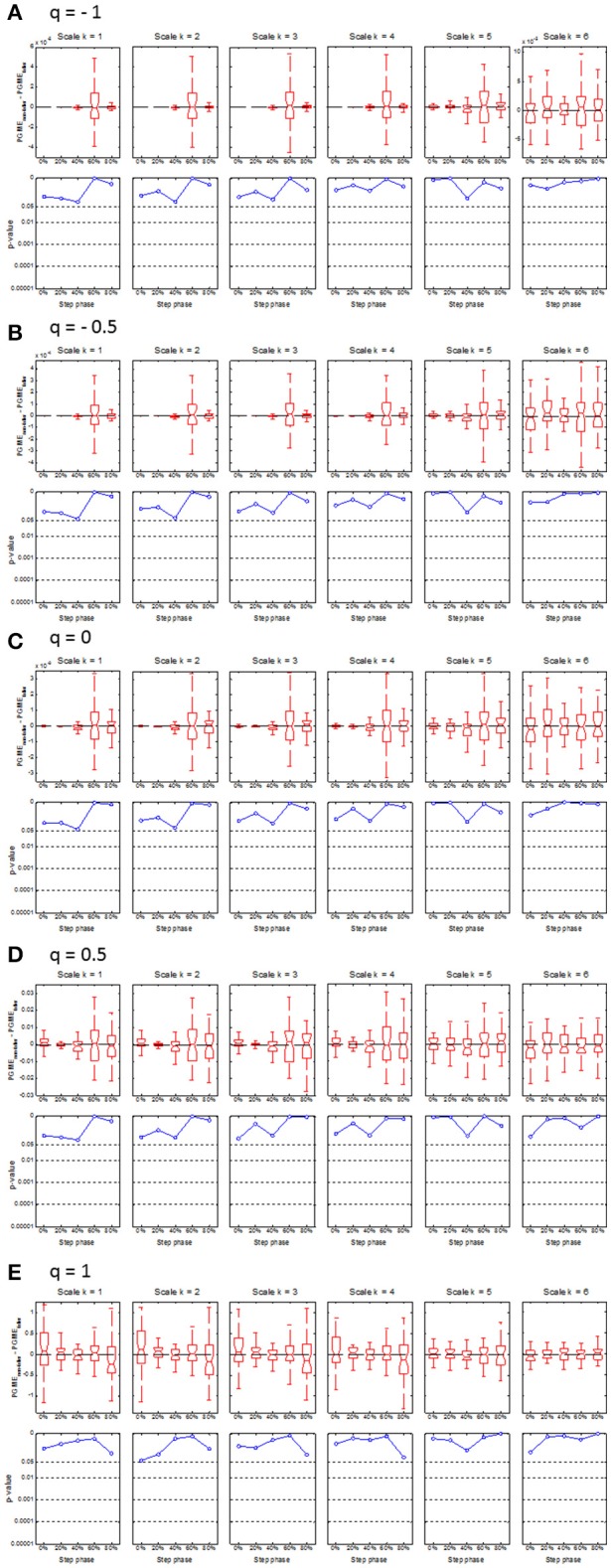
Boxplots (upper row) and corresponding *p*-values (lower row) of the median difference between PGME of multiple-time fallers and their matched non-falling controls. The x-axis of each plot indicates how the median difference or corresponding *p*-values change with the step phase. The upper and lower panels on each row **(A**–**E)** indicate how the median difference and the corresponding *p*-values change with the scale *k* = 1–6. Rows **(A**–**E)** of the panels indicate how the median difference in PGME and corresponding *p*-values change according to q-orders, *q* = −1, −0.5, 0, 0.5, and 1, of the PGME. Note that the center of the boxes represents the group median and the upper and lower borders of the box represent the 75th and 25th percentile, respectively. The whiskers represent the most deviating values within 1.5 times the interquartile range from the median value and the notches represent the confidence interval of the median value.

Compared to matched non-fallers, single-time fallers had significantly (*p* < 0.0001) lower PGME at 60% of the step cycle, which was consistent across *q* = −1 to 0.5 and scale *k* = 1 to 4 (see Figure 3). This difference disappeared for the higher *k* scales and when the *q* value approached *q* = 1, which corresponds with the conventional sample entropy. A significantly (*p* < 0.0004) lower PGME was also found at 40% of the step cycle for *q* between −1 and 0 and scale between 1 and 3, where the differences disappeared for *q* > 0 and scale *k* > 3. No significant differences were found for PGME of multiple-time fallers when compared to the matched non-fallers (see Figure 4).

### Feature selection

For a single fall within the 6-month follow-up period, the predictive ability of PGME at 60% of the step phase at scale *k* = 2 to 4 and *q* = 0.5 to 0.8 was ranked highest in the PLS prediction model by the TP-loadings (see upper part of Table 3). For prediction of a single fall, the PLS feature selection procedure found 2 latent variables for the PGME metrics (see upper part of Table 3), 7 latent variables for the conventional gait features and demographic variables (see upper part of Table 4), and 4 latent variables for all features combined. For prediction of multiple falls within the 6-month follow-up period, the PLS feature selection procedure found 4 latent variables for the PGME metrics (see lower part of Table 3), 7 latent variables for the conventional gait features and demographic variables (see lower part of Table 4), and 3 latent variables for all features combined. The highest ranked PGME metrics for the prediction of single falls were contained in a single PLS latent variable, whereas the highest ranked PGME parameters for prediction of multiple falls were contained in four latent variables (see right column in Table 3). These results suggest that different components of PGME are related to the risk for single and multiple falls within the 6-month follow-up period. The gait features with the highest predictive ability for falls (Table 2) also differed between the prediction of single vs. multiple falls within the 6-month follow-up period, which indicates that different components of trunk acceleration gait are important when predicting single vs. multiple falls (see Table 4). The conventional sample entropy (i.e., *k* = 1 and *q* = 1) was not one of the selected parameter settings in the PLS prediction model, which indicates that the q-orders of PGME improve fall prediction when compared with conventional sample entropy (i.e., PGME for *q* = 1).

**Table 3:** The top 10 ranked parameter settings of the PGME metrics in the PLS prediction model and their latent variable number.

**Rank**	**Phase**	**Scale *k***	***q*-order**	**Latent var #**
**SINGLE-TIME FALL PREDICTION**				
1	60%	2	0.6	1
2	60%	2	0.5	1
3	60%	3	0.7	1
4	60%	4	0.8	1
5	80%	6	0.8	1
6	40%	2	0.7	1
7	40%	2	0.5	1
8	40%	6	1.0	1
9	80%	3	0.5	1
10	60%	3	0.7	1
**MULTIPLE-TIME FALL PREDICTION**				
1	0%	2	0.8	3
2	20%	5	0.8	4
3	60%	6	0.7	3
4	80%	5	0.1	1
5	80%	5	−0.8	1
6	20%	2	−1.0	1
7	0%	5	−0.6	3
8	40%	5	−0.8	2
9	0%	5	−0.1	1
10	60%	3	−0.5	1

*None of the selected PGME were equal to conventional sample entropy (q = 1 and k = 1)*.

**Table 4:** The top 10 ranked gait features and demographic variables in the PLS prediction model and their latent variable number.

**Rank**	**Feature name**	**Feature type**	**Direction**	Latent var #^*^
**SINGLE-TIME FALL PREDICTION**				
1	Harmonic Ratio	Gait	AP	1 (7)
2	Number of falls (past 6 month)	Other	–	2
3	Low freq. percentage	Gait	ML	4
4	Stride time variability	Gait	–	7
5	Acc. standard deviation	Gait	ML	6
6	Stride regularity	Gait	ML	1
7	Stride freq. variability	Gait	AP	1
8	Low freq. percentage	Gait	AP	1
9	Body mass	Other		4
10	Spectral dominant freq.	Gait	AP	1
**MULTIPLE-TIME FALL PREDICTION**				
1	Harmonic ratio	Gait	V	1 (6)
2	Number of falls (past 6 month)	Other	–	5
3	Harmonic ratio	Gait	AP	1
4	Low frequency percentage	Gait	ML	3
5	Spectral freq. range	Gait	ML	3
6	Low frequency percentage	Gait	AP	3
7	Lyapunov W	Gait	AP	1(2)
8	Stride regularity	Gait	AP	1(2)
9	Stride regularity	Gait	ML	1(2)
10	Stride regularity	Gait	V	1(2)

* *Note that values within the brackets indicate that the gait features are equally represented in two latent variables*.

### Fall prediction ability of PGME

The PGME showed an improved predictive ability for a single fall within a 6-month follow-up period when compared to conventional gait features and demographic variables (i.e., feature set 2) (see Table 5). The PGME metrics contained in a single PLS component predicted single falls with an accuracy of 0.76 (95% CI 0.75–0.77), which was significantly better (*p* < 0.001) than conventional gait features and fall history. The ΔAICc and relative likelihood in Table 6 indicate that the model based on PGME metrics only was the preferred model to predict single falls. With an accuracy of 0.68 (95% CI 0.67–0.69), the PGME yielded less accurate predictions of multiple falls compared to its prediction of single falls within the 6-month follow-up period. In contrast, conventional gait features and demographic variables (i.e., features set 2) predicted multiple falls more accurately than single falls, with an accuracy of 0.77 (95% CI 0.76–0.80) (see lower part of Table 5). Nevertheless, when taking into account the number of latent variables, the PLS model based on the PGME metrics had an equal performance to the PLS model based on conventional gait features and demographic variables (*p* = 0.05, see bottom part of Table 6). The best prediction of multiple falls was achieved based on a combination of conventional gait features, demographic variables, and PGME metrics and had an accuracy of 0.83 (95% CI 0.82–0.84).

**Table 5:** Performance of the PLS based single- and multiple time fall prediction model.

	**Sensitivity**	**Specificity**	**Positive predictive value**	**Negative predictive value**	**Accuracy**
**SINGLE TIME FALLERS (*****N*** = **52)**					
PGME	0.71 (0.70, 0.72)	0.80 (0.79, 0.81)	0.79 (0.78, 0.80)	0.74 (0.73, 0.75)	0.76 (0.75, 0.77)
Gait features + Demograph. var	0.61 (0.60, 0.62)	0.76 (0.75, 0.77)	0.73 (0.72, 0.74)	0.66 (0.65, 0.67)	0.68 (0.67, 0.69)
Fall history: 6 months	0.47 (0.46, 0.48)	0.67 (0.65, 0.68)	0.59 (0.57, 0.60)	0.56 (0.55, 0.57)	0.57 (0.56, 0.58)
All combined	0.75 (0.73, 0.76)	0.80 (0.79, 0.81)	0.80 (0.79, 0.81)	0.77 (0.76, 0.78)	0.77 (0.76, 0.78)
**MULTIPLE TIME FALLERS (*****N*** = **46)**					
PGME	0.67 (0.66, 0.69)	0.69 (0.68, 0.70)	0.69 (0.68, 0.70)	0.69 (0.68, 0.70)	0.68 (0.67, 0.69)
Gait features + demograph var	0.79 (0.77, 0.80)	0.76 (0.75, 0.77)	0.77 (0.76, 0.78)	0.79 (0.78, 0.80)	0.77 (0.76, 0.80)
Fall history: 6 months	0.55 (0.53, 0.56)	0.65 (0.64, 0.67)	0.62 (0.61, 0.63)	0.60 (0.59, 0.61)	0.60 (0.59, 0.61)
All combined	0.83 (0.82, 0.84)	0.83 (0.82, 0.84)	0.84 (0.83, 0.85)	0.84 (0.83, 0.85)	0.83 (0.82, 0.84)

**Table 6:** Comparison of difference in AIC (ΔAICc) and relative likelihood (RL) for the three PLS fall prediction models of single-time and multiple-time fallers.

	**PGME**	**Gait features + Demographic var**.	**All combined**
**SINGLE-TIME FALLERS**			
PGME^*^	–	10.37 (0.006)	4.44 (0.11)
Gait features	10.37 (0.006)	–	5.93 (0.05)
All combined	4.44 (0.11)	5.93 (0.05)	–
**MULTIPLE-TIME FALLERS**			
PGME	–	6.02 (0.05)	1.90 (0.39)
Gait features	1.90 (0.39)	–	4.13 (0.13)
All combined^*^	6.02 (0.05)	4.13 (0.13)	–

Note that

* *indicates the best performing model*.

## Discussion

The main aim of the present study was to introduce a new entropy measure of daily-life walking, called phase-dependent generalized multiscale entropy (PGME), that combines nonlinear analysis, spectral decomposition, and phase-dependent analysis of the trunk acceleration data, and to test the predictive ability of PGME with respect to falls in community-dwelling older adults. Our findings of a lower PGME for single-time fallers indicates that this group has less irregular trunk acceleration during gait compared to trunk acceleration of non-fallers at 60% of the step cycle for *q* < 1. The finding of a difference at 60% of the step cycle between single-time fallers and non-fallers is consistent with a previous study indicating that local dynamic stability at 0 and 60% distinguishes retrospective fallers from non-fallers (Ihlen et al., 2015). Furthermore, a lower irregularity of gait dynamics for single-time fallers is consistent with a recent report displaying lower irregularity (i.e., multiscale entropy) of the walking dynamics in elderly fallers when compared with non-fallers (Ihlen et al., 2016a). These studies suggest that differences in the stability and irregularity of trunk dynamics between fallers and non-fallers may be caused by differences in the push-off phase, which takes place at around 60% of the step cycle. In contrast, another study on multiscale entropy found less irregular trunk dynamics in elderly non-fallers compared to fallers (Riva et al., 2013). However, that study used a different state space reconstruction method that separated between AP, ML, and V directions. Furthermore, the lower irregularity for the single-time fallers in the present study was found for *q* < 1 and not for conventional multiscale entropy (i.e., *q* = 1), which indicates that the difference in irregularity between fallers and non-fallers depends on the definition of the entropy metrics. In line with this suggestion, the present study did show a tendency of higher irregularity for non-fallers compared to single-time fallers for *q* = 1, even though this tendency was not significant (see Figure 3E).

Even though the present study found clear differences in PGME between single-time fallers and matched non-fallers, no consistent differences were found between multiple-time fallers and matched non-fallers. Almost half (47.2%) of the single-time prospective fallers had experienced falls within the 6 months prior to the baseline measurements and could therefore be considered as multiple-time fallers when 6 months prospective and retrospective data are combined (van Schooten et al., 2016). Post hoc analyses indicated that single-time fallers with a history of falls had a lower irregularity compared to those with no history of falls (all *p* < 0.005). Interestingly, the lower irregularity was most pronounced at 60% of the stride cycle for the single-time fallers with fall history when compared to non-fallers, whereas the lower irregularity was most pronounced at 40% of the stride cycle for the first-time fallers. This may indicate differences in gait kinematics during ground contact or push-off phase between first-time fallers and single-time fallers with a fall history. Multiple-time fallers with a fall history had significantly higher irregularity (*p* < 0.002) compared to the multiple-time fallers with no fall history, where the difference was most pronounced at 40% of the step cycle and for conventional multiscale entropy (i.e., *q* ≈ 1). Thus, the post-hoc analyses further support the suggestion that the irregularity features of the walking dynamics of community-dwelling older persons are dependent on both fall history and their risk of future falls. This dependency may be related to fear of falling and other variables of functional decline in the older population. However, the relationship between PGME and variables on health status in older adults was not investigated, which might be important to further improve the clinical interpretation of PGME.

There may be other reasons that could explain the difference found between the variables relevant for the prediction of single and multiple falls. The group of multiple-time fallers may contain a higher portion of healthy and active older adults with a higher fall risk due to higher exposure, without having impaired gait stability. The lower irregularity in the first-time fallers could also reflect an adaptation to decrease fall risk, which might not be possible anymore for the multiple fallers. Nevertheless, the added value of PGME to predict falls in combination with other gait features indicates that this metric is a valuable addition to fall prediction models.

The present study introduced a new entropy measure, PGME, and showed its value for prediction of single and multiple falls in community-dwelling older persons within a 6-month follow-up period. However, a few limitations need to be pointed out. First, PGME distinguished between single- and multiple-time fallers and their matched non-fallers based on a relatively small sample of community-dwelling older adults from a single geographical location. The accuracy reported for the prediction of single- and multiple falls within a 6-month follow-up period are in the upper end of values that could be expected from a perfect fall prediction model (Palumbo et al., 2015). Thus, it is likely that accuracy will decrease for PGME and the other gait features for larger samples from multiple locations. Further prospective studies on larger samples from multiple locations are necessary before concluding that PGME will improve individual fall prediction in a global population of community-dwelling older adults.

Secondly, variables of clinical tests used for fall risk assessment, such as tests of balance and mobility performance, were not included in the classification procedure. Inertial sensor-based tools for unsupervised in-home testing of physical function, including mobility and balance, could contribute to the improvement of early fall prediction in community-dwelling older adults (Shany et al., 2012). However, previous studies have shown that features of daily-life walking improve fall prediction when combined with instrumented tests of mobility performance (Weiss et al., 2013). Nevertheless, falls have multifactorial causes including medication, loss of muscle strength, vision, footwear, environmental hazards, cognitive function, mental health, and fear of falling, to mention but a few, and it is therefore likely that a combination of outcomes of clinical tests and features of daily-life activities will optimize fall prediction models. Even though the inclusion of PGME might further improve the fall risk assessment when combined with clinical tests, issues such as cost (and maintenance cost) of accelerometers, unsupervised device handling in an in-home setting, provision and retrieval from patients in a clinical setting, and the potential for an easy-to-use online estimation of PGME may be decisive for the feasibility of the use of PGME in fall risk assessment and fall prediction tools. Thus, further studies and cost-benefit analyses have to be conducted to determine the usability and feasibility of these analyses.

Thirdly, the present study used a PLS regression model for fall prediction. This model was chosen because it has the ability to combine a high number of features into a low number of latent variables to prevent overfitting. The supplementary material (Appendix D) shows the fall prediction ability of a support vector machine (SVM) algorithm with a RELIEFF feature selection. The SVM algorithm had a substantial decrease in its performance for all feature sets when compared to PLS regression. Thus, as confirmed in previous papers from our research group, PLS regression seems to be one of the better performing models for fall risk assessment and fall prediction (Ihlen et al., 2015, 2016b). Further studies could use sparse PLS regression incorporating a matrix regularization procedure in the PLS regression for a more efficient feature selection (Chun and Keles, 2010).

Fourthly, the present study did not investigate walking speed dependency of the PGME, although the difference between single-time fallers and matched non-fallers may be walking speed dependent. Previous studies have found that nonlinear measures are speed dependent and, consequently, the phase-dependency of PGME may change with walking speed (Jordan et al., 2007; Kang and Dingwell, 2008; Bruijn et al., 2009). Although conventional sample entropy has recently been shown not to vary over gait speeds, further studies should investigate the influence of walking speed on PGME (Huijben in revision).

Fifthly, the accuracy of the phase-dependency of PGME is dependent on the reliability of step identification, even more so than conventional gait quality measures. The inertial sensor was placed on the lower back, which complicates the identification of heel strike and toe-off events within the gait cycle. Thus, the phase-dependency PGME in Figures 3, 4 were not defined according to single and double support phases within the gait cycle, but according to the local minima of the vertical trunk velocity. Advanced step identification algorithms might define the phase-dependent PGME according to heel strike and toe-offs, but further validation of these algorithms is necessary (e.g., Godfrey et al., 2015). Furthermore, as inertial sensors become smaller and more wearable, further studies might include additional sensors on the lower extremities and/or insole pressure data to identify heel strikes and toe offs and thereby single and double support phases.

## Conclusion

The present study introduces a new entropy measure of daily-life walking, called phase-dependent generalized multiscale entropy (PGME), and assesses PGME performance in predicting falls in community-dwelling older adults. PGME showed superior performance in predicting a single fall in a 6-month follow-up period. PGME indicated that single-time fallers, but not multiple-time fallers, had significantly lower entropy at 60% of the step cycle when compared with non-fallers. PGME may therefore be a promising metric to improve fall prediction amongst community-dwelling older adults. However, external validation of PGME on larger multi-site samples, and the relationship between PGME and gait speed, health status, and physiological correlates have to be assessed to further support the clinical relevance of this novel metric.

## Author contributions

EI: Conception and design of work, analysis, interpretation of data, revising the manuscript critically for important intellectual content, and final approval of the manuscript. KvS and MP: Collecting data, interpretation of data, revising the manuscript critically for important intellectual content, and final approval of the manuscript. SB and JvD: Analysis, interpretation of data, revising the manuscript critically for important intellectual content, and final approval of the manuscript. JH and BV: Interpretation of data, revising the manuscript critically for important intellectual content, and final approval of the manuscript.

### Conflict of interest statement

The authors declare that the research was conducted in the absence of any commercial or financial relationships that could be construed as a potential conflict of interest.
