# Supplementary material for: Improved Prediction of Falls in Community-Dwelling Older Adults Through Phase-Dependent Entropy of Daily-Life Walking
Source: Front Aging Neurosci. 2018 Mar 5;10:44. doi: 10.3389/fnagi.2018.00044 (PMC5844982; doi:10.3389/fnagi.2018.00044)
Supplement: Supplementary file 1 [file DataSheet1.docx]

**Appendix A
Technical details of the multivariate empirical mode decompositions (MEMD)**

The following MEMD algorithm introduced by Rehman and Mandic (2010) was used in the present study (see acknowledgment):

*Step 1*: Generate a Hammersley sequence-based point set on a 3*m* – 1 dimensional sphere where *m* is the number of lags in the state space reconstruction method (*m* = 1, 2, and 3 in the present study).

*Step 2*: Compute the projection $p_{\theta_{k}}(t)$of the gait dynamics x(t) = (or residual **r**(*t*) or **d**(*t*) for iterative steps) along the unit direction vectors *θ_k_* of the 3*m* – 1 dimensional sphere.

*Step 3*: Find the time instant $t_{\theta_{_{k}}}$that corresponding to the maxima $p_{\theta_{k}}^{\max}(t)$ of $p_{\theta_{k}}(t)$ along all *k* = 1, 2,…,3*m* – 1 dimensions.

*Step 4*: Obtain the envelope curves, $e_{\theta_{k}}(t)$, by component-wise spline interpolations between all time instants $t_{\theta_{_{k}}}$of $p_{\theta_{k}}^{\max}(t)$.

*Step 5*: Compute the mean **m**(*t*) of all envelope curves, $e_{\theta_{k}}(t)$, across all 3*m* – 1 directions of the sphere by the following equation:

$\mathbf{m}(t)=\frac{1}{3m-1}\sum_{k=1}^{3m-1} e_{\theta_{k}}(t)$ (A6)

*Step 6*: The first series of details **d**_1_(*t*) around the mean **m**_1_(*t*) is defined as **d**_1_(*t*) = **x**(*t*) – **m**_1_(*t*). If **d**_1_(*t*) satisfies the selected stopping criteria, then **d**_1_(*t*) is defined as an intrinsic mode function (IMF) and Step 2 to 5 is performed on first residual, **r**_1_(*t*) = **x**(*t*) – **d**_1_(*t*). The second IMF is defined as **d**_2_(*t*) = **r**_1_(*t*) – **m**_2_(*t*) with residual **r**_2_(*t*) = **r**_1_(*t*) – **d**_2_(*t*). Consequently, the *n*th IMF is defined as **d***_n_*(*t*) = **r***_n_*_-1_(*t*) – **m**_n_(*t*) with residual **r***_n_*(*t*) = **r***_n_*_-1_(*t*) – **d***_n_*(*t*). This iterative shifting procedure (i.e., Step 2 to 5) is continued until two maxima $p_{\theta_{k}}^{\max}(t)$of the projection$p_{\theta_{k}}(t)$ in Step 3 can no longer be found. If **d***_n_*(*t*) does not satisfy the stopping criteria, step 2 to 5 are performed as an iterative procedure on **d***_n_*(*t*) until the stopping criteria is met and an IMF is defined. Subsequently, steps 2 to 5 are repeated on the residual, **r***_n_*(*t*) = **r***_n_*_-1_(*t*) – **d***_n_*(*t*). The stopping criteria used in the present study is similar to the stopping criteria proposed by Rilling et al. (2003), except that we in/excluded the criteria of equality between the number of zero crossings and number of maxima. The sum of all IMFs and the final residual, $\mathbf{x}(t)=\sum_{n=1}^{N} \mathbf{d}_{n}(t)+\mathbf{r}_{N}(t)$, correspond to the gait dynamics **x**(*t*).

**Appendix B**

Generalized sample entropy (i.e., qSaEn) for the trunk dynamics in reconstructed state space (see Eq. 1 and 2 in the main text) were defined by the following equation (Silva and Murta, 2012):

$qSaEn_{r}=\log_{q}(n_{x})-\log_{q}(n_{y})$ (B1)

where the *n_x_* and *n_y_* are the total normalized number of points in state space with distance below r of the reconstructed gait dynamic of Eq. 1 and Eq. 2, respectively, in the main text. *n_x_* and *n_y_* across *j* points is given by:

$n_{x}=\frac{1}{N-l}\sum_{j=1}^{N-l} n_{j,x}$ and $n_{y}=\frac{1}{N-2l}\sum_{j=1}^{N-2l} n_{j,y}$ (B2)

whereas *n_j_,_x_* and *n_j_,_y_* is given by the following two equations:

$n_{j,x}=\frac{1}{N-l}\sum_{j=1}^{N-l} \Theta\left(d\left[X_{j},X_{i}\right]\right)$ for *i* ≠ *j* (B3)

$n_{j,y}=\frac{1}{N-2l}\sum_{j=1}^{N-2l} \Theta\left(d\left[Y_{j},Y_{i}\right]\right)$ for *i* ≠ *j* (B4)

where *N* is the sample size of the 3D acceleration or velocity signal, *l* is the lag size, and $d\left[X_{j},X_{i}\right]$ is the Chebyshev distance between two points of the gait dynamics *X_j_* and *X_i_* defined by Eq. 1 or 2 above. The Heavyside step function $\Theta(d)$ is given by the following equation:

$\Theta(d)=\left\{\begin{matrix} 0,\text{ }d\geq r \\ 1,\text{ }d<r \end{matrix}\right.$ (B5)

The computation of qSaEn differs from the conventional computation of sample entropy because the points *X_j_* and *X_i_* of the gait dynamics in Eq. B3 and B4 are defined as points in the reconstructed gait dynamics from 3D acceleration (i.e., Eq. 1 and 2 in the main text) whereas the conventional SaEn is based on the dynamics reconstructed from 1D signals.

**Appendix C**

**Matlab code for the phase-dependent generalized multiscale entropy (PGME)**

The following Matlab function, PGME_compute, estimates PGME as a function of scale, q-order and phase within the step cycle. The Matlab code calls the function, phase_qSaEn, to calculate the phase-dependent *q*-order sample entropy and memd developed by Silva and Murta (2012), which is available at http://www.commsp.ee.ic.ac.uk/~mandic/research/emd.htm.

function [PGME]=PGME_compute(TempMat1,TempMat2,locs,r,phase,q,scl)

%Matlab function to estimate phase-dependent generalized multiscale entropy (PGME)

%Computation time: 135 sec for length(TempMat) = 3000 on a Xeon 2.8 GHz CPU

%Input%%%%%%%%%%%%%%%%%

%___TempMat1__________Reconstructed state space of dimension m (e.g. Eq. 1)

%___TempMat2__________Reconstructed state space of dimension m+1 (e.g. Eq. 2)

%___locs______________Vector of sample number for the initiation of each step cycle

%___r_________________Threshold r for the Heavy-side step function in Eq. B5

%___phase_____________vector of phases within the step interval (values [0 to 1])

%___q_________________vector of q-orders in Eq. 5

%___scl_______________Number of scales from memd (Eq.3) to be used to compute PGME

%Output%%%%%%%%%%%%%%%%%

%__PGME_______________Phase-dependent generalized multiscale entropy

%--------------------------------------------------------------------------

%Created by Espen A.F. Ihlen ©

%Email: [espen.ihlen@ntnu.no](mailto:espen.ihlen@ntnu.no)
%Please refer to the paper: Improved prediction of falls in community-dwelling %older adults through phase-dependent irregularity of daily-life walking
%The authors take no responsibility in the use of this Matlab function

%------Code----------------------------------------------------------------

% Multivariate Empirical Mode Decomposition of reconstucted state space;

% TempMat1 and TempMat2; see Appendix A

PGME=zeros(scl,length(q),length(phase));

imf1=memd(TempMat1);

imf2=memd(TempMat2);

%Computation of generalized sample entropy (qSaEn) with respect to step-phase and scale

for n=1:length(phase),%Step-phase

locs2=locs(1:end-1)+round(phase(n).*diff(locs));%location in the step cycle

for nn=1:scl, %Scale

%Low-pass filtered TempMat1 (see Eq. 3)

X1_imf(1:size(imf1,1),1:size(imf1,3))=sum(imf1(:,nn:end,:),2);

%Low-pass filtered TempMat2 (see Eq. 3)

X2_imf(1:size(imf2,1),1:size(imf2,3))=sum(imf2(:,nn:end,:),2);

%Computation of phase-dependent qSaEn (see Appendix B)

[phase_qsaen] = Phase_qSaEn(X1_imf',X2_imf',locs2,r,0.1,q);

%Phase-dependent generalized multiscale entropy (PGME)

PGME(nn,1:length(q),n)=phase_qsaen;

end

end

function [phase_qsaen] = Phase_qSaEn(tempMat1,tempMat2,locs,r,prc,q)

%Matlab function estimate phase-dependent generalized sample entropy

%Input%%%%%%%%%%%%%%%%%

%___tempMat1__________Reconstructed state space of dimension m (e.g. Eq. 1)

%___tempMat2__________Reconstructed state space of dimenion m+1 (e.g. Eq. 2)

%___locs______________Vector of sample number for the initiation of each step cycle

%___r_________________Threshold r for the Heavy-side step function in Eq. 9

%___prc_______________Portion/interval (value between 0 and 1) after starting point used to compute phase_qSaEn

%___q_________________vector of q-orders to compute phase_qSaEn

%Output%%%%%%%%%%%%%%%%%

%__phase_saen_________Phase-dependent qSaEn

%-------------------------------------------------------------------------

%Created by Espen A.F. Ihlen ©

%Email: [espen.ihlen@ntnu.no](mailto:espen.ihlen@ntnu.no)
%Please refer to the paper: Improved prediction of falls in community-dwelling %older adults through phase-dependent irregularity of daily-life walking
%The authors take no responsibility in the use of this Matlab function

%------Code----------------------------------------------------------------

tempMat1=tempMat1';

tempMat2=tempMat2';

maxiter=round(prc.*diff(locs));

count1=zeros(size(tempMat1));

count2=zeros(size(tempMat2));

logq1=zeros(size(q));

logq2=zeros(size(q));

phase_qsaen=zeros(size(q));

%Phase-dependent counts (see Eq. B2-B5) for reconstructed state space of Eq. 1

for i = 1:length(tempMat1)

% calculate Chebyshev distance, excluding self-matching case

dist1 = max(abs(tempMat1(:,i+1:length(tempMat1)) - repmat(tempMat1(:,i),1,length(tempMat1)-i)));

D1 = (dist1 < r);

count1(i) = sum(D1)/length(tempMat1);

end

count1_phase=0;

length1_phase=0;

for i=1:length(locs)-1

length1_step=length(locs(i):locs(i)+maxiter(i));

length1_phase=length1_phase+length1_step;

count1_phase=sum(count1(locs(i):locs(i)+maxiter(i)))+count1_phase;

end

%Phase-dependent counts (see Eq. B2-B5) for reconstructed state space of Eq. 2

for i = 1:length(tempMat2)

% calculate Chebyshev distance, excluding self-matching case

dist2 = max(abs(tempMat2(:,i+1:length(tempMat2)) - repmat(tempMat2(:,i),1,length(tempMat2)-i)));

D2 = (dist2 < r);

count2(i) = sum(D2)/length(tempMat2);

end

count2_phase=0;

length2_phase=0;

for i=1:length(locs)-1,

length2_step=length(locs(i):locs(i)+maxiter(i));

length2_phase=length2_phase+length2_step;

count2_phase=sum(count2(locs(i):locs(i)+maxiter(i)))+count2_phase;

end

%Computation of phase-dependent q-order sample entropy (see Eq. 4 and Eq. B1)

correl1 = count1_phase/length1_phase;

correl2 = count2_phase/length2_phase;

for nq=1:length(q),

logq1(nq)=((correl1^(1-q(nq)))-1)/(1-q(nq));

logq2(nq)=((correl2^(1-q(nq)))-1)/(1-q(nq));

if q(nq)==1,

phase_qsaen(nq)=log(correl1/correl2);

else

phase_qsaen(nq)=logq1(nq)-logq2(nq);

end

end

%--------------------------------------------------------------------------

**Appendix D**

**Supplementary material**

The PLS regression model used in present study was compared with a support vector machine prediction model based on ReliefF procedure (see Kira and Rendell, 1992 for further details). SVM is a binary classifier that will try to create a *m*-1 dimensional boundary in a *m* dimensional feature space that optimizes the separations of fallers and non-fallers (Cortes and Vapnik, 1995). Similar to the PLS prediction model, the most influential PGME metrics in the SVM prediction model were defined at 60% of the step cycle whereas the influence of scale *k* = 4 and *q* = -0.5 to 0.4 seemed to be different from the PLS prediction model (see Table A1). The gait features identified as most influential by SVM differed from the PLS prediction model (compare Table A2 with Table 4 in the main text). However, the SVM prediction model supported the findings of the PLS prediction model that different features are selected for prediction of single- and multiple-time fallers. The SVM prediction model had a substantially lower accuracy for both single- and multiple-time fallers when compared to the PLS prediction model (compare Table A3 and Table 5 in the main text). The ReliefF feature selection procedure is not integrated in the SVM and, thus, may choose a sub-optimal combination features for the SVM prediction model.

**Table A1:** The top ten ranked parameter settings of the PGME metrics in the ReliefF feature selection and SVM model.

| Rank | Phase | Scale *k* | *q*-order |
| --- | --- | --- | --- |
| Single-time fall prediction | | | |
| 1 | 60 % | 4 | 0.1 |
| 2 | 60 % | 4 | 0.4 |
| 3 | 60 % | 4 | -0.4 |
| 4 | 60 % | 4 | 0 |
| 5 | 60 % | 4 | -0.3 |
| 6 | 60 % | 4 | -0.1 |
| 7 | 60 % | 4 | 1 |
| 8 | 80 % | 6 | 0.2 |
| 9 | 60 % | 4 | -0.5 |
| 10 | 60 % | 4 | -0.2 |
| : | : | : | : |
| 115 | 60% | 1 | 1 |
| Multiple-time fall prediction | | | |
| 1 | 60% | 6 | 0.4 |
| 2 | 60% | 6 | 0.6 |
| 3 | 60% | 6 | 0.5 |
| 4 | 80% | 4 | 0.9 |
| 5 | 60% | 6 | 0.3 |
| 6 | 80% | 4 | 1.0 |
| 7 | 60% | 6 | 0.7 |
| 8 | 60% | 6 | 0.2 |
| 9 | 80% | 4 | 0.8 |
| 10 | 60% | 6 | 0.1 |
| : | : | : | : |
| 145 | 20% | 1 | 1 |

**Table A2:** The top ten ranked gait features and demographic variables in the ReliefF feature selection and SVM model.

| Rank | Feature name | Feature type | Direction |
| --- | --- | --- | --- |
| Single-time fall prediction | | | |
| 1 | Lyapunov exponent RC | Gait | V |
| 2 | Lypunov per stride RC | Gait | V |
| 3 | Harmonic ratio | Gait | V |
| 4 | Lyapunov RC | Gait | AP |
| 5 | Frequency range (Weiss) | Gait | ML |
| 6 | Harmonic RatioP | Gait | V |
| 7 | Standard deviation | Gait | ML |
| 8 | Lyapunov per stride W | Gait | ML |
| 9 | Lyapunov RC | Gait | AP |
| 10 | Index harmonicity | Gait | AP (4) |
| Multiple-time fall prediction | | | |
| 1 | Harmonic ratio P | Gait | V |
| 2 | Harmonic ratio P | Gait | AP |
| 3 | Dominant Freq (Weiss) | Gait | V (3) |
| 4 | LASA fall risk score | Other | -- |
| 5 | Stride frequency | Gait | -- |
| 6 | Stride time | Gait | -- |
| 7 | Harmonic Ratio | Gait | V |
| 8 | Harmonic Ratio | Gait | AP |
| 9 | Dominant Freq (Weiss) | Gait | AP |
| 10 | Low freq precentage | Gait | AP |

**Table A3**: Performance of ReliefF feature selection and support vector machine (SVM).

|  | Sensitivity | Specificity | Pos. predictive value | Neg. predictive value | Accuracy |
| --- | --- | --- | --- | --- | --- |
|  | *Single time fallers (N = 52)* | | | | |
| PGME | 0.65 (0.64, 0.66) | 0.67 (0.66, 0.68) | 0.67 (0.66,0.68) | 0.67 (0.66,0.68) | **0.66 (0.65,0.67)** |
| Gait features + demograph. var | 0.55 (0.54, 0.56) | 0.55 (0.53,0.56) | 0.55 (0.54, 0.56) | 0.55 (0.54, 0.56) | **0.55 (0.54, 0.56)** |
| Fall history:  6 months | 0.47 (0.46,0.48) | 0.67 (0.65,0.68) | 0.59 (0.57,0.60) | 0.56 (0.55,0.57) | **0.57 (0.56,0.58)** |
| All combined | 0.51 (0.49,0.52) | 0.54 (0.53, 0.56) | 0.53 (0.52,0.54) | 0.53 (0.53, 0.54) | **0.53 (0.52, 0.54)** |
|  | *Multiple time fallers (N = 46)* | | | | |
| PGME | 0.66 (0.65, 0.68) | 0.64 (0.62, 0.65) | 0.66 (0.65, 0.67) | 0.66 (0.65, 0.67) | **0.65 (0.64,0.66)** |
| Gait features + demograph var | 0.69 (0.68, 0.71) | 0.69 (0.68, 0.71) | 0.71 (0.70, 0.72) | 0.71 (0.70, 0.72) | **0.69 (0.68, 0.70)** |
| Fall history:  6 months | 0.55 (0.53,0.56) | 0.65 (0.64,0.67) | 0.62 (0.61,0.63) | 0.60 (0.59,0.61) | **0.60 (0.59, 0.61)** |
| All combined | 0.67 (0.66, 0.68) | 0.70 (0.68, 0.71) | 0.70 (0.69, 0.71) | 0.69 (0.68,0.70) | **0.68 (0.67,0.69)** |
